# Determinants of late-onset neonatal sepsis among neonates admitted to the neonatal intensive care unit of Arba-Minch general hospital, southern Ethiopia

**DOI:** 10.1371/journal.pone.0279622

**Published:** 2022-12-30

**Authors:** Alemseged Aydiko, Teklemariam Gultie, Gossa Fetene Abebe, Temesgen Ginbeto, Gebresilasea Gendisha Ukke

**Affiliations:** 1 Department of Midwifery, College of Medicine and Health Sciences, Mizan-Tepi University, Mizan Teferi, Ethiopia; 2 Department of Midwifery, College of Medicine and Health Sciences, Arba-Minch University, Arba Minch, Ethiopia; 3 School of Public Health, College of Medicine and Health Sciences, Mizan-Tepi University, Mizan Teferi, Ethiopia; 4 Health Systems and Equity, Eastern Health Clinical School, Monash University, Box Hill, Victoria, Australia; WCU: Wachemo University, ETHIOPIA

## Abstract

**Introduction:**

Neonatal sepsis can be either early (<7 Days) or late-onset ≥7days) neonatal sepsis depending on the day of the occurrence. Despite the decrement in early onset neonatal sepsis, there is still an increment in late-onset neonatal sepsis. Ethiopian demography and health survey report showed an increment in neonatal mortality in 2019/20.

**Objective:**

The objective of this study was to assess the determinants of late-onset neonatal sepsis among neonates admitted to the neonatal intensive care unit at Arba-Minch general hospital, southern Ethiopia.

**Methods:**

An institution based study was conducted from March 1, 2021, to June 30, 2021 in Arba-Minch general hospital. Cases were neonates diagnosed with late-onset neonatal sepsis with their index mother chart and controls were neonates admitted with other diagnoses at the same period. Cases and controls were selected consecutively. Data extraction tool and interview which was developed by reviewing different kinds of literature was used to collect data. Data were entered by using Epi data version 3.1software and transformed to Statistical Package for Social Sciences version 25 software for analysis. The binary logistic regression model was used to assess determinants and variables with a p-value <0.2 were transformed to multivariable logistic regression then, a p-value < 0.05 with 95% confidence interval were used to declare significant association with the outcome variable.

**Result:**

A total of 180 subjects (60 cases and 120 controls) were included in this study. The mean age of neonates was 12.1 days with standard deviations of 4.3. Multivariable logistic regression analysis showed that; history of either sexually transmitted disease /urinary tract infection [AOR = 9.4; 95%CI(3.1–28.5)], being preterm (gestational age of <37 weeks) [AOR = 4.9; 95%CI (1.7–13.7)], use of endotracheal intubation/mechanical intubation [AOR = 8.3; 95%CI (1.8–26.4)]and either mixed types of infant feeding option or formula feeding before admission [AOR = 12.7; 95%CI(3.7–42.8)]were significantly associated with late-onset neonatal sepsis.

**Conclusion and recommendations:**

This study revealed that antenatal, intrapartum and postpartum factors have shown an association with late-onset neonatal sepsis. It is recommended to strengthen counseling and advice to mothers with specific risk factors of late-onset neonatal sepsis.

## Introduction

Neonatal sepsis is a systemic infection occurring in infants within 28 days of life and is a major cause of morbidity and mortality in newborns [1]. It’s is defined as systemic inflammatory response syndrome in the presence of or as a result of suspected or proven infection in a neonate. The infection could be of bacterial, viral, fungal, or rickettsial origin, according to the international pediatric sepsis consensus conference of 2005 [2].

Neonatal sepsis encompasses various systemic infections of the newborn such as septicemia, meningitis, pneumonia, arthritis, osteomyelitis, etc., but it does not include superficial infections like thrush [3].

Based on the onset age of the disease, neonatal sepsis is divided into early onset neonatal sepsis or late-onset sepsis. Early neonatal sepsis (EOS) is mainly due to organisms acquired before and during delivery (or maternal-fetal infection), whereas late-onset neonatal sepsis (LONS) is due to organisms acquired after delivery. However, there is little consensus as to what age limits apply, with early onset ranging from 48 hours to 7 days after delivery [4].

Neonatal sepsis arises when pathogenic microorganisms gain entry into the bloodstream causing devastating systemic infection within the first 28 days of life [5]. There are a number of risk factors associated with neonatal sepsis. Common risk factors for neonatal sepsis in sub-Saharan Africa have been identified as prematurity, premature rupture of membranes (PROM), maternal pyrexia, low birth weight and obstructed labor and birth asphyxia which is usually characterized by low first and fifth Apgar scores at birth [6].

Late-onset neonatal sepsis (LONS) is caused by the organisms thriving in the external environment of the home or the hospital. The infection is often transmitted through the hands of the care providers. The onset of symptoms is usually delayed beyond 72 hours after birth and the presentation is that of septicemia, pneumonia, or meningitis. LONS is a common complication of prolonged admission to the neonatal intensive care unit (NICU) following preterm birth [7].

It’s known that newborns are the future generation, so ensuring the health, growth and development of them through all the activities should be the primary concern of every society [8]. These newborns are susceptible and vulnerable to morbidity and mortality. Even if there is a relatively rapid decrement in under-five mortality, neonatal mortality remains still high [9].

Early onset neonatal sepsis is mainly related to maternal factors by which the neonate acquires infections from the mother either prior to or during childbirth, while late-onset of neonatal sepsis is mainly related with the post-natal related things which have potential relation with the newborn after delivery [10].

Globally, there is a decrement in the incidence of early onset neonatal sepsis but the studies show this figure is opposite for the incidence of late-onset of neonatal sepsis in the last ten years [11].

Sub-Saharan Africa has a high burden of neonatal mortality, leading to an estimated 49.6% of all under-five deaths in 2013 [12]. In Ethiopia, approximately 42% of the under-5 mortality is mainly related to neonatal mortality [13]. According to the 2016 Ethiopia Demographic and Health Surveys (EDHS), the country is experiencing a high neonatal mortality rate at 29 per 1000 live births but there is a slight increment compared to Ethiopian 2019 mini Demographic and Health Surveys in which it is about 30 per 1000 live births [14].

The determinants of neonatal mortality are not well documented and stated in Ethiopia, but the studies conducted previously reported different determinants of neonatal mortality and additionally the determinants of late-onset of neonatal sepsis are not well stated and differentiated in Ethiopia [15]. Also, they are not well documented in the specific study area.

The primary Beneficiary of the study will be neonates who will receive treatment from ArbaMinch general hospital and the community under the catchment area. Once the determinants of late-onset of neonatal sepsis are determined stakeholders can initiate different programs and projects by considering those determinants.

This study has also benefits to ArbaMinch general Hospital, policymakers, non-governmental organizations working on this specific area, and the zonal health department by addressing the specific determinants of late-onset of neonatal sepsis.

So, the objective of this study was to assess the determinants of late-onset neonatal sepsis among neonates admitted to the neonatal intensive care unit of Arbaminch general hospital, southern Ethiopia.

## Methods and materials

### Study area and period

This study was conducted in Arbaminch general hospital, which is the only general hospital in Gamo zone. Arba Minch General hospital is situated in Arbaminch town, which is located about 500 kilometer (km) to the south of Addis Ababa, the capital city of Ethiopia and 280 Km from Hawassa, the center of the southern nation’s nationality and people regional state (SNNPR). The town has 1 general hospital, 1primary hospital, 2 health centers and more than 30 private health institutions which are giving service to almost 4 million people. Currently, Arbaminch general hospital is providing the healthcare services to 3.8 million people. Among them, 884,733 are females under the reproductive age group (15–49) and 112,774 are infants. The hospital has a total of 266 beds and the neonatal intensive care unit has 20 beds. The last 3-month hospital report shows there have been 352 admissions to the neonatal intensive care unit. The study was conducted from March 1, 2021 to June 30, 2021.

### Study design

An institutional based case-control study design was employed.

### Population

#### Source population

All neonates, who were admitted to the neonatal intensive care unit of Arbaminch general hospital.

#### Study population

All neonates, who were admitted to the neonatal intensive care unit of Arbaminch general hospital from March 1, 2021, to June 30, 2021.

**Cases** are among those who are admitted to the NICU and diagnosed as having late-onset neonatal sepsis by general practitioners or senior specialists according to the guideline with the established integrated management of newborn and childhood illness (IMNCI) guideline.

**Controls** are all other neonates who have the diagnosis other than late-onset neonatal sepsis.

### Study participants

Neonates who were admitted to the neonatal intensive care unit of Arbaminch general hospital from March 1, 2021, to June 30, 2021, and selected during the study period.

### Eligibility criteria

#### Inclusion criteria

All neonates admitted to the NICU of ArbaMinch general hospital and diagnosed as having late-onset neonatal sepsis by a physician from March 1, 2021 to June 30, 2021.

### Sample size determination

A two population proportion formula (using OpenEpi, Version 3) was used to estimate the sample size required for the study by considering that the proportion of mothers with intra partum fever among the controls of 4.5% (main exposure variable), which was estimated from another study (16), 95% Confidence interval, 80% power of the study, case to control ratio of 1:2 to detect an odds ratio of 5.5 which was estimated from a study done by Gebremedhin D, Berhe H, Gebrekirstos K. [16]. Then the sample size was 162, by adding 10% non-respondent rate a total sample size of 180 (60 cases and 120 controls) was the estimated sample size for this study.

### Sampling procedure

Among the neonates who were admitted to the NICU of ArbaMinch general hospital from March 1, 2021 to June 30, 2021, both the cases and controls were selected consecutively depending on the time of admission.

### Study variables

#### Dependent variable

- Late-onset neonatal sepsis

#### Independent variables

- **Socio-demographic characteristics of the neonate and index mother are**:-age, marital status, religion, ethnicity, residence, education level, occupation, monthly income, age of neonate and sex of neonate.
- **Maternal health-related Characteristics are**:- Parity, Antenatal care, place of delivery, mode of delivery, assistant during delivery, duration of labor, intra-partal fever, Premature rupture of membrane, hypertensive disorder during pregnancy, Antepartum hemorrhage and history of Urinary tract infection/Sexually transmitted infections.
- **Breastfeeding related Characteristics are**:- Breastfeeding initiation time and type of breastfeeding before admission and
- **Neonatal health-related Characteristics are**:—Gestational age, 1^st^ and 5^th^ minute Apgar score, birth weight, immediate crying after birth, resuscitation and endotracheal intubation/mechanical ventilation.

#### Operational definition

- **Late-onset neonatal sepsis**: If sepsis has occurred within 7 and 28 days of age which is diagnosed by the general practitioner and senior specialist at ArbaMinch General Hospital according to the guideline [17].

### Data collection tool and procedure

Data were collected by using a Data extraction tool and interview. The tool was developed after reviewing different kinds of literature related to the title [16–22]. The data extraction tool has medical record number, socio-demographic characteristics of neonate and index mother and neonatal, maternal and breastfeeding health-related characteristics. The data were collected at NICU of Arbaminch general hospital, after the neonate were diagnosed as having late onset neonatal sepsis, the subject was taken as case, then a mother of the neonates was interviewed and after the interview checklist was filled. Then following the case, the consecutive 2 neonates without late onset neonatal sepsis were taken as controls and similarly checklist was filled after interviewing the mothers. Face to face interview was used to collect data from the mother of neonates. Data was collected by 5 data collectors having Bachelor of sciences in midwifery and it were supervised by 1 Masters of sciences student each day. Before the collection training was given to the data collectors on how to record and extract/collect data.

### Data quality control

In order to assure the quality of the data, from the beginning, the tool was developed after reviewing different literatures. As well the formal communication was made with the chief executive officer (CEO), medical director and matron of the hospital and the objective of the study was clearly stated and discussed with them. The training for data collectors was also aimed at assuring the quality of data. Every day after the collection of data discussion was made among the data collector and principal investigator to check the correctness and fullness of data. Data cleaning was made after entry to assure the quality of data.

### Data processing and analysis

Data were checked for completeness and then coded, entered and cleaned by using Epi data version 3.1software. And then it was exported to Statistical Package for Social Sciences (SPSS) version 25 software for analysis. The descriptive statistics with cross-tabulation was used to present the finding of the study. The Chi-square test was done initially and the variables which met the chi-square assumption were entered into the bivariate analysis. The association between the outcome variable (late-onset neonatal sepsis) and several independent variables were analyzed in the Binary logistic regression model. Then, variables having a p-value < 0.2 in binary logistic regression were retained and entered into the multivariable logistic regression analysis. Multi-collinearity was checked by using correlation coefficient and variance inflation factors and interactions were assessed among independent variables. Age of the mother and neonatal crying at birth were not included in the model as they were highly correlated with parity and resuscitation at birth. The degree of association between outcome and independent variables was determined by using OR with a CI of 95% and p-value. A p-value < 0.05 was considered as a cutoff point to declare that there is a statistically significant association between the dependent and independent variables. Model fitness was checked by using Hosmer and Lemeshow test and the value was 0.872.

### Ethical consideration

The ethical clearance was obtained from Arbaminch University, College of medicine and health sciences IRB (Institutional Review Board) with a reference number of IRB/1065/21. Then, a written formal letter which was obtained from Arbaminch University, College of medicine and health sciences was submitted to the hospital and concerning bodies.

Before the collection of the data, oral consent was obtained from the mothers of the neonates after describing the objective of the study. The study subject benefited from the study as the identification of specific determinants is helpful during an intervention in the specific problem. This study may have no potential risk on both the mother and neonate and the information from the subjects was kept confidential since the questionnaire has code.

## Results

### Socio-demographic characteristics of the respondents

A total of 180 participants were included in this study. Among them, 60 were neonates who had late-onset neonatal sepsis (cases) with their index mother and 120 were neonates who had no late-onset neonatal sepsis (controls) with their index mother and making the response rate of 100%. The mean age of the mothers was 26.46 years with a standard deviation of 5.59 years and it ranges from 17 to 40 years. The median income of participants was 4000 Ethiopian Birr ($76) (with inter-quartile range of 1750–8000 Ethiopian Birr ($33- $152). Among the mothers who participated in the study, 80% (48) of the cases and 79.2% (95) of the controls were married and 8.3% (5) of the case and 14.2% (17) of controls were single by marital status. Regarding residence, 66.7% (40) of the cases and 66.7% (80) of the controls were living in Urban. Twenty-five (41.7%) of cases and 25% (30) of controls were housewives by occupation and 25% (15) of cases and 33.3% (40) of controls had an educational status of college and above. Regarding the socio-demographic characteristics of the neonate, the mean age of the neonates was 12.1 days with a standard deviation of 4.3 and with regard to sex proportion of neonates, males were higher in cases 39(65%) than controls of 70(58.3%) (Table 1).

**Table 1:** Socio-demographic characteristics of the neonates and their mothers included in the study.

Variables	Category	Cases n = 60(%)	Controls n = 120(%)	Total n = 180(%)
**Mother’s age**	15–19	6(10%)	14(11.7%)	20(11.1%)
	20–24	18(30%)	34(28.3%)	52(28.9%)
	25–29	19(31.7%)	39(32.5%)	58(32.2%)
	30–34	12(20%)	21(17.5%)	33(18.3)
	35 and Above	5(8.3%)	12(10%)	17(9.4%)
**Marital status**	Single	5(8.3%)	17(14.2%)	22(12.2%)
	Married	48(80%)	95(79.2%)	143(79.4%)
	Others*	7(11.7%)	8(6.7%)	15(8.3%)
**Religion**	Orthodox	28(46.7%)	59(49.2%)	87(48.3%)
	Protestant	23(38.3%)	45(37.5%)	68(37.8%)
	Others**	9(15%)	16(13.3%)	25(13.9%)
**Ethnicity**	Gamo	30(50%)	65(54.2%)	95(52.8%)
	Wollaita	9(15%)	21(17.5%)	30(16.7%)
	Goffa	8(13.3%)	10(8.3%)	18(10%)
	Konso	6(10%)	6(5%)	12(6.7%)
	Oromo	3(5%)	7(5.8%)	10(5.6%)
	Amhara	4(6.7%)	11(9.2%)	15(8.3%)
**Residence**	Urban	40(66.7%)	80(66.7%)	120(66.7%)
	Rural	20(33.3%)	40(33.3%)	60(33.3%)
**Maternal education**	No formal education	14(23.3%)	17(14.2%)	31(17.2%)
	Primary (Grade 1^st^ -8^th^)	9(15%)	24(20%)	33(18.3%)
	Secondary(Grade 9^th^ -10^th^)	12(20%)	19(15.8%)	31(17.2%)
	Grade 11^th^– 12^th^	10(16.7%)	20(16.7%)	30(16.7%)
	College and higher	15(25%)	40(33.3%)	55(30.6%)
**Occupation of mother**	Housewife	25(41.7%)	30(25%)	55(30.6%)
	Civil servant	17(28.3%)	29(24.2%)	46(25.6%)
	Business woman	10(16.7%)	26(21.7%)	36(20%)
	Private organization	4(6.7%)	6(5%)	10(5.6%)
	Others***	4(6.6%)	29(24.2%)	33(18.3%)
**Neonate’s sex**	Male	39(65%)	70(58.3%)	109(60.6%)
	Female	21(35%)	50(41.7%)	71(39.4%)

**Key**:—Others* = Divorced, separated and widow

Others** = Musilm and catholic

Others*** = Daily laborers and students

### Maternal obstetric (pregnancy, labor and delivery) history of the respondents

More than half of the mothers, 24(52.2%) of cases and 61(56.5%) of controls had ANC visits four times and 14(23.3%) of the case and 12(10%) of controls had no ANC visit during their pregnancy. Thirty-six (60%) of cases and 67(55.8%) of controls were multiparous by parity and the mean parity for cases was 2.3(±1.42) and controls were 2.41(±1.49) with a minimum of 1 and maximum of 7. Concerning the mode of delivery 27(45%) 0f the cases and almost half, 59(49.2%) of the controls had a spontaneous vaginal delivery. Regarding PROM 26(43.3%) of cases and 21(17.5%) of controls had a history of premature rupture of the membrane during labor (Table 2).

**Table 2:** Obstetric characteristics of mothers of the neonates.

Variables	Category	Cases n = 60(%)	Controls n = 120(%)	Total n = 180(%)
**ANC follow-up**	Yes	46(76.7%)	108(90%)	154(85.6%) 26(14.4%)
	No	14(23.3%)	12(10%)	
**N****o** **of ANC vist**	1 visit	4(8.7%)	1(0.9%)	5(3.2%)
	2 visit	4(8.7%)	14(13.0%)	18(11.7%)
	3 visit	14(30.4%)	32(29.6%)	46(29.9%)
	4 visit	24(52.2%)	61(56.5%)	85(55.2%)
**Parity**	Primipara	20(33.3%)	45(37.5%)	65(36.1%)
	Multiparous	36(60%)	67(55.8%)	103(57.2%)
	Grand multiparous	4(6.7%)	8(6.7%)	12(6.7%)
**Place of delivery**	Home	14(23.3%)	7(5.8%)	21(11.7%)
	Health institution	46(76.7%)	113(94.2%)	159(88.3%)
**Mode of delivery**	SVD	27(45%)	59(49.2%)	86(47.8%)
	Instrumental delivery	16(26.7%)	25(20.8%)	41(22.8%)
	C/S	17(28.3%)	36(30%)	53(29.4%)
**Assistant during delivery**	Health professionals	44(73.3%)	114(95%)	158(87.8%)
	Others	16(26.7%)	6(5%)	22(12.2%)
**Duration of labor**	≤ 18hr	44(73.3%)	105(87.5%)	149(82.8%)
	>18hr	16(26.7%)	15(12.5%)	31(17.2%)
**History of PROM**	Yes	26(43.3%)	21(17.5%)	47(26.1%)
	No	34(56.7%)	99(82.5%)	133(73.9%)
**Duration of PROM**	≤ 12hr	17(65.4%)	21(100%)	38(80.9%)
	> 12hr	9 (34.6%)	0(0%)	9(19.1%)
**Intra-partal fever**	Yes	12(20%)	8(6.7%)	20(11.1%)
	No	48(80%)	112(93.3%)	160(88.9%)
**History of anyHypertensive disorder of pregnancy**	Yes	9(15%)	24(20%)	33(18.3%)
	No	51(85%)	96(80%)	147(81.7%)
**History of any APH**	Yes	6(10.0%)	15(12.5%)	21(11.7%)
	No	54(90.0%)	105(87.5%)	159(88.3%)
**History of UTI/STI**	Yes	23(38.3%)	14(11.7%)	37(20.6%)
	No	37(61.7%)	106(88.3%)	143(79.4%)

**Key**:—Others = Traditional birth attendants, Relatives and health extension workers

### Neonatal characteristics of the neonates

In this study, the mean (±SD) gestational age at the time of delivery of the neonates was 36.67±2.12 weeks for the cases and 38.12 ± 2.64 weeks for controls. Twenty (36.4%) of cases and 88(73.3%) of controls were term during the delivery and 43(93.5%) of cases and almost two-third of controls, 73(63.5%) had normal birth weight. Regarding Apgar scores more than half of cases and controls, 25(61%) and 62(54.9%) respectively had Apgar scores of less than 7 in the 1^st^ minute (Table 3).

**Table 3:** Neonatal characteristics of the neonates admitted to NICU of Arbaminch general hospital, Gamo zone, Ethiopia.

Variables	Category	Cases n = 60(%)	Controls n = 120(%)	Total n = 180(%)
**Gestational age**	<37wk(preterm)	35(63.6%)	32(26.7%)	67(38.3%)
	≥37wk(term)	20(36.4%)	88(73.3%)	108(61.7%)
**Birth Weight**	<2500gm(low birth weight)	3(6.5%)	42(36.5%)	45(28%)
	≥2500gm(normal birth weight)	43(93.5%)	73(63.5%)	116(72%)
**1**^**st**^ **Min APGAR score**	<7	25(61%)	62(54.9%)	87(56.5%)
	≥7	16(39%)	54(47.8%)	70(43.5%)
**5**^**th**^ **Min APGAR score**	<7	5(12.2%)	6(5.3%)	11(7.1%)
	≥7	36(87.8%)	107(94.7%)	143(92.9%)
**Neonates cried at birth**	Yes	39(65%)	95(79.2%)	134(74.4%)
	No	21(35%)	25(20.8%)	46(25.6%)
**Resuscitation at birth**	Yes	21(35%)	24(20%)	45(25%)
	No	39(65%)	96(80%)	135(75%)
**Endotracheal intubation/Mechanical ventilation**	Yes	13(21.7%)	8(6.7%)	21(11.7%)
	No	47(78.3%)	112(93.3%)	159(88.3%)

### Breastfeeding related characteristics of cases and controls

**Fig 1:**
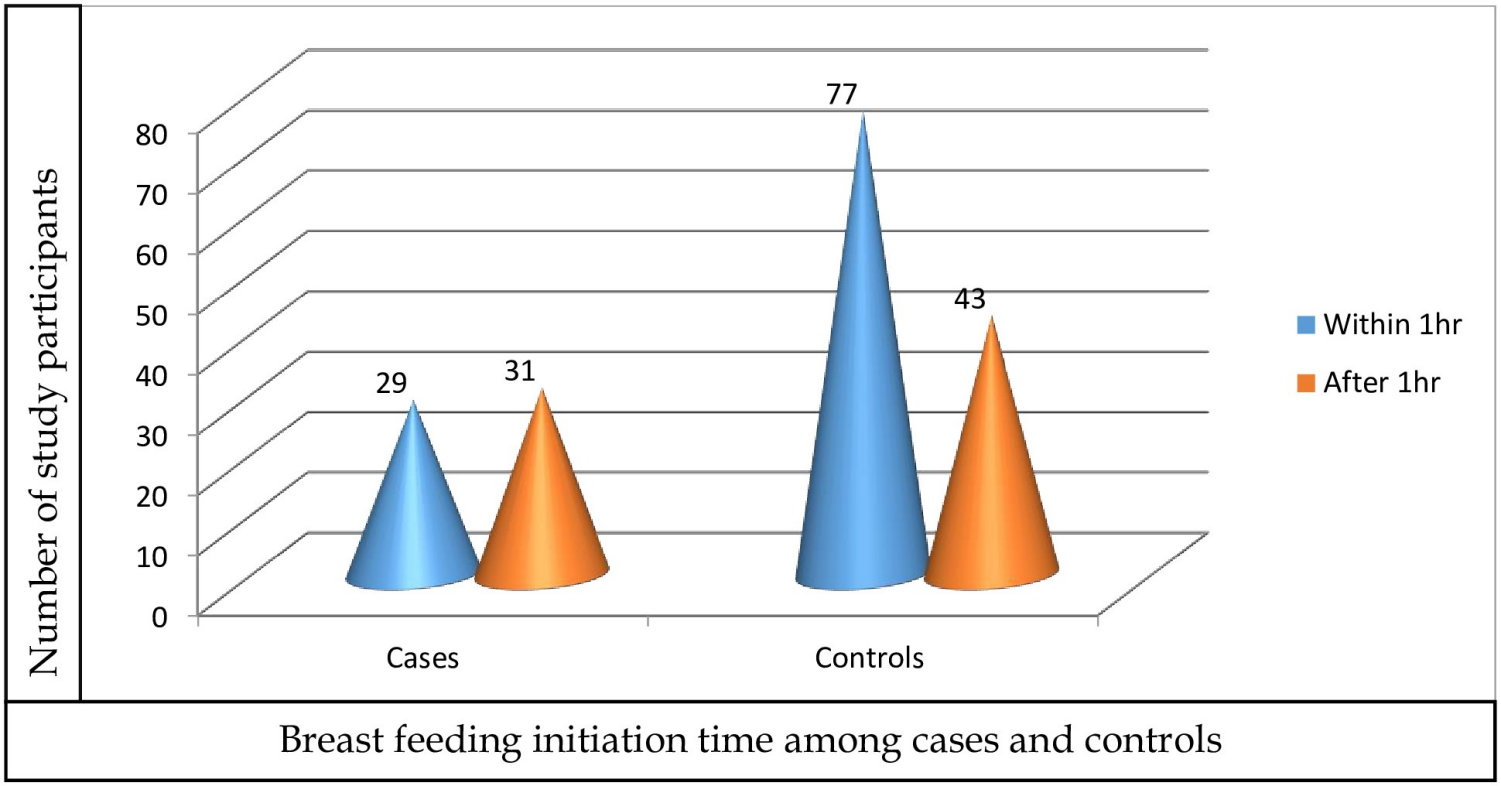
Breastfeeding initiation time among the study participants admitted to NICU of Arbaminch general hospital, Gamo zone, Ethiopia.

Regarding breastfeeding initiation time, the proportion of those who initiated breastfeeding within one hour was higher in controls, 77(64.2%) when compared with cases, 29(48.3%) as shown in the graph below (Fig 1).

**Fig 2:**
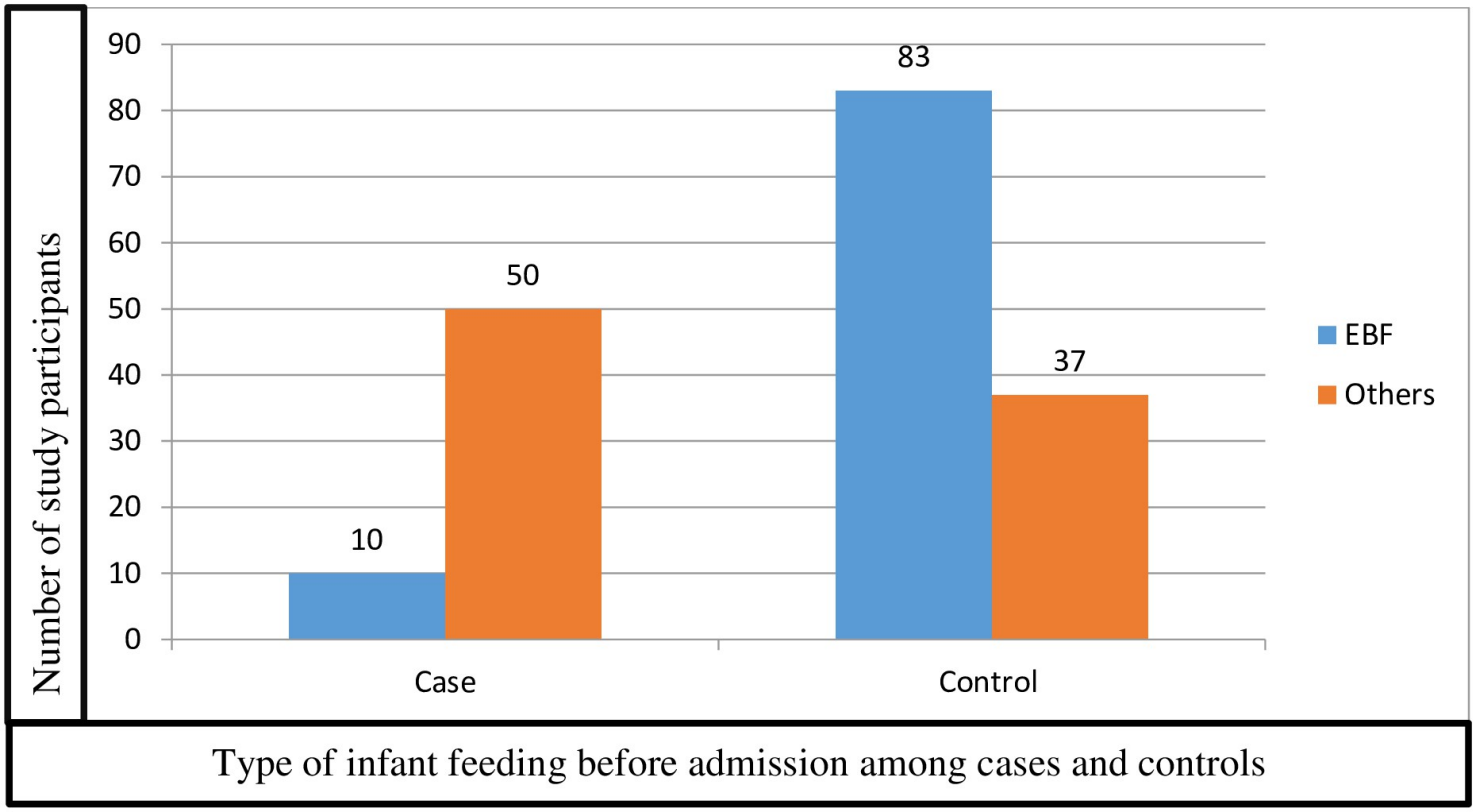
Infant feeding options among neonates admitted to NICU of Arbaminch general hospital, Gamo zone, Ethiopia. **Key**:—Others = Mixed type and formula feeding.

This study revealed, 83(69.2%) of the controls used exclusive breastfeeding as an option of infant feeding type and, 50(83.3%) of the cases were practicing mixed type or formula as an option of breastfeeding as shown in the figure below (Fig 2).

### Determinants of late-onset neonatal sepsis

As can be noted from the result of bivariate analysis at a p-value of < 0.2, out of 24 maternal and neonatal variables 14 variables had a statistically significant association with late-onset neonatal sepsis. Which were; maternal educational level, occupation of the mother, ANC follow-up, place of delivery, duration of labor, intra-partal fever, STI/UTI, breastfeeding initiation time, gestational age, 5^th^ min APGAR score, Assistant during delivery, resuscitation at birth, endotracheal intubation and types of breastfeeding before admission.

Multivariable logistic regression was done by taking the variables which were statistically significant in bivariate logistic regression (p-value of <0.2) into account simultaneously. Then conditional back-ward elimination regression method was used after checking multi co-linearity.

In the multivariable logistic regression analysis, four variables had shown an overall significant association with late-onset neonatal sepsis at p-value less than 0.05 level of significance.

History of either STI/UTI during index pregnancy had shown significant association with late-onset neonatal sepsis. The odds of neonates having late-onset neonatal sepsis among neonates, whose mothers had a history of either STI/UTI during the index pregnancy was 9.4 times higher when compared to neonates whose mothers didn’t have a history of STI/UTI in the index pregnancy [AOR = 9.4; 95%CI(3.1–28.5)].

Gestational age had also shown a significant association with late-onset neonatal sepsis. The odds of neonates having late-onset neonatal sepsis who were born preterm (before 37 weeks) is 4.9 times higher when compared with those neonates who were born at term (at or after 37 weeks) [AOR = 4.9; 95%CI (1.7–13.7)].

Endotracheal intubation/mechanical ventilation during resuscitation had also shown significant association with late-onset neonatal sepsis. Those neonates who had either endotracheal intubation/mechanical ventilation during resuscitation had 8.3 times higher odds of developing late-onset neonatal sepsis when compared to those neonates who had no endotracheal intubation during resuscitation [AOR = 8.3; 95%CI (1.8–26)].

Type of infant feeding before admission had shown significant association with late-onset neonatal sepsis. Those neonates who had either mixed type of feeding or formula feeding before admission were 12.75 times higher odds of developing late-onset neonatal sepsis when compared to those neonates who had exclusive breastfeeding before admission [AOR = 12.7; 95%CI(3.7–42.8)] (Table 4).

**Table 4:** Bivariate and multivariable logistic regression analysis result of determinant variables of late-onset neonatal sepsis admitted to NICU of Arbaminch general Hospital, Gamo zone, Ethiopia.

Variables	Category	Cases n = 60(%)	Controls n = 120(%)	COR [95% CI]	AOR [95% CI]
**Maternal education**	No formal education	14(23.3%)	17(14.2%)	2.8(0.8–5.5)*	0.794(0.136–4.654)
	Primary	9(15%)	24(20%)	1(0.37–2.63)	0.951(0.208–4.337)
	Secondary	12(20%)	19(15.8%)	1.68(0.66–4.3)	0.16(0.029–0.879)
	Grade 11^th^–12^th^	10(16.7%)	20(16.7%)	1.33(0.5–3.5)	0.26(0.049–1.39)
	College and higher	15(25%)	40(33.3%)	1	1
**Occupation of mother**	Housewife	25(41.7%)	30(25%)	1	1
	Civil servant	17(28.3%)	29(24.2%)	0.703(0.3–1.56)	1.09(0.161–7.517)
	Business woman	10(16.7%)	26(21.7%)	0.46(0.18–1.13)*	1.157(0.189–7.088)
	Private organization	4(6.7%)	6(5%)	0.8(0.2–3.15)	0.934(0.039–22.638)
	Others^**1**^	4(6.6%)	29(24.2%)	0.166(0.051–0.53)*	0.177(0.02–1.542)
**ANC follow-up**	Yes	46(76.7%)	108(90%)	1	1
	No	14(23.3%)	12(10%)	2.739(1.177–6.375)*	2.141(0.184–24.922)
**Place of delivery**	Home	14(23.3%)	7(5.8%)	4.913(1.863–12.95)*	0.6(0.13–2.6)
	Health institution	46(76.7%)	113(94.2%)	1	1
**Assistant during delivery**	Health professionals	44(73.3%)	114(95%)	1	1
	Others^**2**^	16(26.7%)	6(5%)	6.909(2.54–18.793)*	6.502(0.36–99.75)
**Duration of labor**	≤ 18hr	44(73.3%)	105(87.5%)	1	1
	>18hr	16(26.7%)	15(12.5%)	2.545(1.16–5.594)*	1.477(0.302–6.932)
**Intra-partal fever**	Yes	12(20%)	8(6.7%)	3.5(1.345–9.108)*	1.145(0.087–15.149)
	No	48(80%)	112(93.3%)	1	1
**History of UTI/STI**	Yes	23(38.3%)	14(11.7%)	4.707(2.19–10.09)*	9.44(3.119–28.59)**
	No	37(61.7%)	106(88.3%)	1	1
**BF initiation time**	≤ 1hr	29(48.3%)	77(64.2%)	1	1
	> 1hr	31(51.7%)	43(35.8%)	1.914(1.021–3.59)*	0.74(0.18–3.031)
**Gestational age**	<37wk(preterm)	35(63.6%)	32(26.7%)	4.813(2.43–9.521)*	4.903(1.74–13.77)**
	≥37wk(term)	20(36.4%)	88(73.3%)	1	1
**5**^**th**^ **min APGAR**	<7	5(12.2%)	6(5.3%)	2.477(0.713–8.6)*	1.922(0.199–18.562)
	≥7	36(87.8%)	107(94.7%)	1	1
**Resuscitated at birth**	Yes	21(35%)	24(20%)	2.154(1.08–4.31)*	1.659(0.386–7.136)
	No	39(65%)	96(80%)	1	1
**Endotracheal Intubation**	Yes	13(21.7%)	8(6.7%)	3.872(1.58–9.956)*	8.308(1.89–26.4)**
	No	47(78.3%)	112(93.3%)	1	1
**Type of infant feeding**	EBF	10(16.7%)	83(69.2%)	1	1
	Others^**3**^	50(83.3%)	37(30.8%)	11.21(5.133–24.51)*	12.75(3.79–42.8)**

**Key** *p-value < 0.2 significant in bivariate analysis and **p-value <0.05 significant in the multivariable analysis. COR- Crude odds ratio, AOR- Adjusted odds ratio, 1-Reference group, Others1 = Daily laborer and students, others2 = Health extension workers, relatives and traditional birth attendant and others3 = mixed and formula feeding

## Discussion

This institutional case-control study has attempted to determine maternal, neonatal and breastfeeding-related determinants of late-onset neonatal sepsis in the NICU of Arbaminch general hospital, Sothern Ethiopia.

This study revealed that the neonates who were born from the mothers who had a history of either STI/UTI during the index pregnancy were 9.44 times more likely to develop late-onset neonatal sepsis compared to those who had no history of either STI/UTI during the index pregnancy. The finding is similar to the studies conducted in Bishoftu, Debrezeyit- Ethiopia [18], Jinka, Southern Ethiopia [19] and study conducted in Mekelle, Northern Ethiopia [16]. This finding is also in line with the study conducted in India and Al-Nasiriayh city, Southern Iraq [20]. This is might be due to the colonization of microbes to the birth canal secondary to urinary and genital tracts and initially those neonates may be discharged with having only prophylactic antibiotics and they may develop sepsis in the late neonatal period.

Another variable that had a significant association with late-onset neonatal sepsis in this study was preterm birth. The neonates who were born preterm (before 37 completed weeks) were 4.93 times more likely to develop late-onset neonatal sepsis when compared to those neonates who were born term (at or after 37 completed weeks). This finding is in line with studies conducted in Temeke and Mwananyamala Hospitals in Dare Salaam, Tanzania [21] to assess the prevalence and associated factors of neonatal sepsis and south-eastern Mexico [22]. This may be due to it’s obvious that the neonates who were born preterm were at risk of developing all neonatal complications especially due to their prematurity they are more at risk of developing late-onset neonatal sepsis at the late-neonatal period due to the prematurity of their immunity response at neonatal period.

Twenty percent of neonates having late-onset neonatal sepsis had had endotracheal intubation or mechanical ventilation after the delivery and the neonates who had endotracheal intubation/mechanical ventilation were 8.308 times more likely to develop late-onset neonatal sepsis when compared to those who didn’t have endotracheal intubation or mechanical ventilation in this study. This finding is similar to the study conducted in Shashamane town, Oromia regional state, Ethiopia [23] and a follow-up study conducted in South-eastern Mexico [22] to risk factors and prognosis of neonatal sepsis. This may be due to the introduction of foreign materials into the mouth and throat of the neonate may results in the colonization by a microorganism that may develop sepsis in the late neonatal period.

Type of infant feeding before admission showed significant association with late-onset neonatal sepsis, neonates who had either mixed type of infant feeding or formula feeding before admission were 12.7 times more likely to develop late-onset neonatal sepsis when compared to those neonates who had exclusive breastfeeding before admission. This may be due to mixed types of breastfeeding is high risk for inflammation of the stomach this results in a possible source of colonization by pathogens and fastens the development of sepsis in the late neonatal period.

### Limitations of the study

Since this study was conducted in the hospital, the result might not be generalizable to the entire population under the catchment area.

This study is quantitative, it was better if mixed method with qualitative approach was also employed to further investigate in detail extra determinants of late-onset neonatal sepsis.

Since the gold standard laboratory investigation for neonatal sepsis is culture, it’s better if the study is conducted in institutions having culture.

## Conclusion

This study found different maternal and neonatal determinants of late-onset neonatal sepsis. The identified possible risk factors of late-onset neonatal sepsis in this study were:—History of either STI/UTI during the index pregnancy, gestational age, endotracheal intubation/Mechanical ventilation and type of breastfeeding before admission.

Based on the findings of this study the following recommendations were forwarded:-

- It’s vital to do ante-natal screening of STI and UTI among the mothers and ensure treatment during pregnancy according to the guideline to decrease its impact on the newborn.
- It’s important to empower health professionals to tackle pre-term labor, prematurity and their complications.
- Postnatal counseling especially regarding exclusive breastfeeding options and the care given to the neonates by health professionals would be better according to the post-natal care guideline.
- This study also recommends further studies to be done by other researchers to address other aspects of determinant factors of late-onset neonatal sepsis with other study designs like Cohort to establish stronger association at the community and institutional level.

## Abbreviations

ANC: Ante-natal care
AOR: Adjusted odds ratio
APGAR: Activity, Pulse, Grimace, Appearance and Respiration
APH: Ante-partum Hemorrhage
BSc: Bachelor of Science
CI: Confidence interval
COR: Crude odds ratio
EBF: Exclusive breastfeeding
EDHS: Ethiopian Demographic and Health Survey
EOS: Early Onset Sepsis
ESR: Erythrocyte sedimentation rate
GA: Gestational Age
HDP: Hypertensive disorder of pregnancy
KMC: Kangaroo Mother Care
LONS: Late-Onset Neonatal Sepsis
NICU: Neonatal Intensive Care Unit
PROM: Premature Rupture of Membrane
SPSS: Statistical Package for Social Science
STI: Sexually Transmitted Infections
UTI: Urinary Tract Infection
WHO: World Health Organization
